# COVID-MENTA: an integrated mental health protection system for pandemic frontline healthcare workers

**DOI:** 10.1192/j.eurpsy.2022.364

**Published:** 2022-09-01

**Authors:** I. Szendi Md Habil, O. Bóna, T. Jenei, C. Kovács, Á. Nagy, K. Németh-Rácz, I.A. Török, E. Rudics, V. Dalos, V. Bilicki, M. Bácsfalvi, K. Téglás, Z. Szabó, E. Kelemen

**Affiliations:** 1 Kiskunhalas Semmelweis Hospital, University Teaching Hospital, Psychiatry Unit, Kiskunhalas, Hungary; 2 University of Szeged, Department Of Psychiatry, Szeged, Hungary; 3 University of Szeged, Department Of Software Development, Szeged, Hungary

**Keywords:** proactive, psychophysiology, Distress, Suicide

## Abstract

**Introduction:**

At Kiskunhalas Semmelweis Hospital, a special mobile container hospital was set up to care for patients infected with SARS-CoV-2 during the first wave of the pandemic.

**Objectives:**

We aimed to create a proactive integrated mental health protection system for the frontline healthcare workers that provides an opportunity for psychophysiological monitoring of stress and crisis during shifts, as well as providing staff with more lasting methods of coping with difficulties.

**Methods:**

From the ascending branch of the second wave, every two weeks on the workers’ rest day, mental helpers initiated a phone call to each employee participating in the program. If it was necessary, we provided psychological counseling, crisis intervention, brief psychotherapy, and psychopharmacotherapy. In addition, self-operated psychophysiological screening devices were used at the frontline work site, which provided an opportunity for continuous telemedicine monitoring.

**Results:**

In our department, three psychologists and three psychiatrists kept in touch with an average of 150 frontline workers per month. Interventions were needed for a total of over 24% in December and January, over 17% in February and March, almost 9% in April, and only 4% in May. Helpers rated an average of two-thirds of these cases as moderate. They faced severe stress 2-3 times a month in sum, and for 2-3 workers needed medication.

**Conclusions:**

Without a mental support system, self-report-based data suggest that nearly half of responders working at the frontline reached the threshold of clinically significant mental syndromes (Greenberg et al, 2021). Using our mental health support system, one-fifth of the workers needed intervention.

**Disclosure:**

No significant relationships.

